# Three-Dimensional Imaging-Guided Lung Anatomic Segmentectomy: A Single-Center Preliminary Experiment

**DOI:** 10.3390/medicina59122079

**Published:** 2023-11-26

**Authors:** Giorgio Cannone, Vincenzo Verzeletti, Alberto Busetto, Luigi Lione, Alessandro Bonis, Samuele Nicotra, Alessandro Rebusso, Marco Mammana, Marco Schiavon, Andrea Dell’Amore, Federico Rea

**Affiliations:** Thoracic Surgery Unit, Department of Cardiac, Thoracic, Vascular Sciences and Public Health, University of Padua, 35122 Padua, Italy; vverzeletti@gmail.com (V.V.); alberto.busetto@aopd.veneto.it (A.B.); luigi.lione@aopd.veneto.it (L.L.); alessandro.bonis@aopd.veneto.it (A.B.); samuele.nicotra@aopd.veneto.it (S.N.); alessandro.rebusso@aopd.veneto.it (A.R.); marco.mammana@aopd.veneto.it (M.M.); marco.schiavon@unipd.it (M.S.); andrea.dellamore@unipd.it (A.D.); federico.rea@unipd.it (F.R.)

**Keywords:** three-dimensional reconstruction, lung cancer, VATS segmentectomy

## Abstract

*Background and objectives*: VATS segmentectomy has been proven to be effective in the treatment of stage I NSCLC, but its technical complexity remains one of the most challenging aspects for thoracic surgeons. Furthermore, 3D-CT reconstruction images can help in planning and performing surgical procedures. In this paper, we present our personal experience of 11 VATS anatomical resections performed after accurate pre-operative planning with 3D reconstructions. *Materials and methods*: A 3D virtual model of the lungs, airways, and vasculature was obtained, starting from a 1.25 mm 3-phase contrast CT scan, and the original images were used for the semi-automatic segmentation of the lung parenchyma, airways, and tumor. *Results*: Six males and five females were included in this study. The median diameter of the pulmonary lesion at the pre-operative chest CT scan was 20 mm. The surgical indication was confirmed in seven patients: in three cases, a lobectomy, instead of a segmentectomy, was needed due to intraoperative findings of nodal metastasis. Meanwhile, only in one case, we performed a lobectomy because of inadequate surgical resection margins. Skin-to-skin operative average time was 142 (IQR 1-3 105–182.5) min. The median post-operative stay was 6 (IQR 1-3 3.5–7) days. The mean value of the closest surgical margin was 13.7 mm. *Conclusion*: Image-guided reconstructions are a useful tool for surgeons to perform complex resections in order to spare healthy parenchyma and to ensure disease-free margins. Nevertheless, human skill and surgeon experience still remain fundamental for the final decisions regarding the proper resection to perform.

## 1. Introduction

Lung cancer is the second most prevalent cancer diagnosis and the leading cause of cancer-related death worldwide. A few years ago, the majority of lung cancers were discovered in advanced stages, where surgery could not determine any indication and patients were referred to a chemotherapy or chemo-radiotherapy program with a dismal prognosis. However, the recent introduction of the Lung Cancer Screening Program and the improvement of radiological items with the spread of Low-dose Computed Tomography machines are making possible the diagnosis of Non-Small Cell Lung Cancer (NSCLC) in its early stages. Unless lung cancer treatment is extremely varied, surgical resection remains the mainstay for early stage and even for locally advanced disease [1,2]. Video-Assisted Thoracoscopic Surgery (VATS) has radically changed the world of thoracic surgery; it has achieved optimal results compared to open approaches [3]. In fact, it ensures less post-operative pain, and it is associated with a reduction in chest tube duration and length of hospital stay (LOS), thus reducing patient morbidity and mortality. For a long time, lobectomy has represented the gold standard for the treatment of all potentially resectable lung tumors, while segmentectomy was considered a lung-sparing option in cases where lobectomy was not feasible because of the patient’s fragility. Nowadays, pulmonary lobectomy and segmentectomy still represent the two main types of anatomical resections for primitive tumors. In particular, segmentectomy has recently been demonstrated to be a valuable alternative to lobectomy, not only in unfit patients. In fact, it has achieved great results in terms of local disease control and disease-free survival for tumors less than 20 mm in diameter [4,5,6]. Moreover, it has demonstrated an acceptable intra and post-operative complications rate. However, it is well-accepted that anatomical segmentectomy is a more challenging procedure compared with lobectomy, because the anatomy of lung segments is more complex. Indeed, it requires a careful assessment of each patient’s anatomy, which may have numerous variations and anomalies in bronchovascular structures. Fortunately, the advent of new radiological techniques and the development of computed tomography (CT) scans with thin sections have allowed surgeons to plan procedures by analyzing radiological images. Some of these new radiological techniques make it possible to create three-dimensional (3D) images starting from CT scan sections. Three-dimensional CT imaging is a powerful tool for thoracic surgeons because it allows us to determine pulmonary anatomy in a more accurate manner when compared to conventional CT images. Moreover, it helps surgeons to better understand the pulmonary anatomy of each patient before and during surgical procedures [7,8,9,10,11,12].

Within this study, we present our data concerning eleven intentional segmentectomies (both complex and simple) performed after an accurate pre-operative evaluation of 3D CT-reconstruction images. The main aim of this preliminary report is to demonstrate the accuracy of this method in the correct identification of bronchovascular structures that may help surgeons during associated procedures. As a secondary goal, we also analyzed the surgical resection margins achieved with the aid of 3D images.

## 2. Materials and Methods

### 2.1. Creating the 3D Model and Pre-operative Evaluations

First, 3D virtual models of the lungs, airways, and vasculature were obtained starting from a 1.25 mm 3-phase contrast CT scan and the resulting Digital Imaging and Communications in Medicine (DICOM) file. The DICOM was processed first using open-source software for image-filtering and preprocessing (3D Slicer) and then with a commercially available software suite for clinical grade image segmentation (Materialise Mimics Innovation Suite 23, Materialise NV, Leuven, Belgium).

The original images were used for the semi-automatic segmentation of the lung parenchyma, airways, and tumor. To obtain a more detailed reconstruction of the internal vessels of the lung, Hessian-based vesselness filtering and adaptive histogram equalization filters were applied to enhance the contrast of those structures [13].

**Figure 1:**
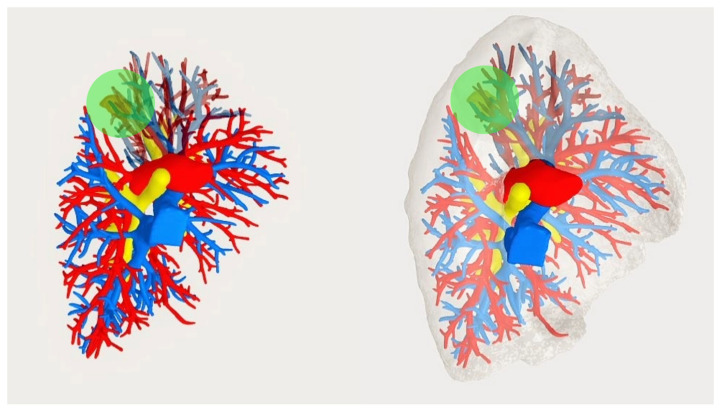
Patient #1: Example of 3D reconstruction of the broncho-vascular anatomy. The airways are shown in yellow, the arteries in red, and the veins in blue. The pulmonary lesion localized in the apico-posterior (S1 + 2) segment of the left upper lobe is depicted in orange, and the safety cuff for the resection margins in green.

**Figure 2:**
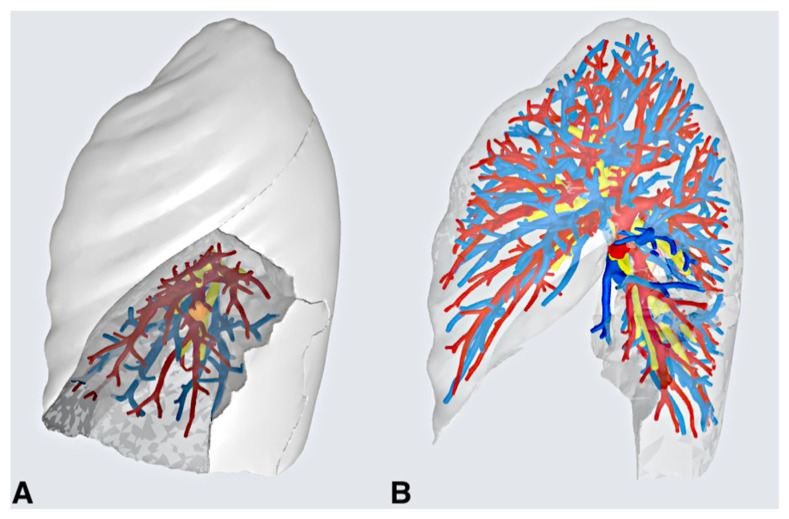
Patient #3. Panel (**A**) highlights the lung parenchyma corresponding to the segments to be resected (7 + 8) along with the nodule and specific broncho-vascular elements. Panel (**B**) simulates the surgical field after parenchymal resection and selective ligation of broncho-vascular elements.

The resulting segmentation of these structures originated from the original dataset and was merged with vessel segmentation from the filtered images. The final segmentation of the whole lung and its surrounding structures was thus obtained. Each segmented anatomical structure was provided for clinical visualization as a stereolithography (.*stl*) file and visualized through an interactive 3D Portable Document Format (PDF) assigning a specific color code to each different anatomical structure (Figure 1). To better navigate the 3D model during surgery, each major branch origin on the 3D model was created independently in order to allow the surgeons to selectively exclude and hide the branches that were surgically ligated during surgery (Figure 2).

Once the virtual 3D model of the patient’s anatomy was ready, it was integrated with other standard exams for surgical planning.

### 2.2. Surgical Procedure

**Figure 3:**
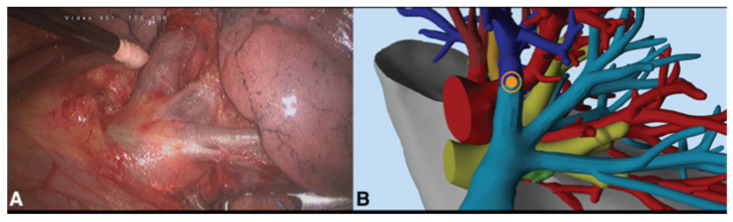
Patient #4: Venous drainage of the left upper pulmonary lobe seen after surgical dissection and isolation of anatomic elements (**A**) and directly from virtual 3D reconstruction (**B**).

The procedure was performed with the patients under general anesthesia with double lumen endotracheal tube with single lung ventilation and placed in a lateral decubitus position. For all patients, we chose a three port VATS anterior approach according to Copenhagen through a 10 mm 3D camera. During surgery, the 3D models were constantly visualized on a dedicated application in order to guide the surgeon’s activity and confirm that the intraoperative anatomy overlapped with our 3D model (Figure 3). In particular, the vascular and bronchial structures of the 3D model could be hidden or enhanced according to the phase of the surgical procedure.

At the end of the operation, the surgical specimens were sent to the laboratories of the Pathological Anatomy Unit for diagnosis.

### 2.3. Variables and Statistics

The following variables were analyzed: gender, age, comorbidities (analyzed with the Charlson Comorbidity Index), history of smoking (former smoker, current smoker, or never smoker), pack/year value (P/Y), tumor dimension, pulmonary lobar localization, surgical indication, type of intervention, number of stapler charges used, final pathological diagnosis, resection margin, skin to skin operative time, presence of adherences, total liquid leaks from the chest tube, presence and days of duration of air leaks, chest drainage duration, day of dismissal, rate of conversion, complications (calculated with the Clavien-Dindo score), and thirty-day mortality. In order to describe nominal variables, we used percentages, whereas we expressed continuous variables with mean, median, standard deviation, and interquartile ranges.

## 3. Results

A total of eleven patients (six females and five males) with a mean age of 63.7 ± 10.3 years (IQR1-3 59.5–68.5) were included in this study. The mean Charlson Comorbidity Index of the patients was 5.5 (IQR1-3 5–7). Two patients were smokers at the time of surgery, while 5 were former smokers. Median pack per year consumption was 17 (Table 1). Five patients suffered from comorbid pulmonary diseases, of whom two suffered from chronic obstructive pulmonary disease (COPD), one from tuberculosis disease (TBC), and two from previous contralateral lung carcinomas.

The median diameter of pulmonary lesions in the preoperative chest CT-scan images was 20 mm (IQR1-3 17–23 mm). For all eleven patients, 3D reconstructions were obtained through the above presented method, with a total time of three working days. The scheduled segmental resections, according to the preoperative evaluation, and those performed are summarized in Table 1. Surgical indication was confirmed in seven patients. In three cases, a lobectomy, instead of a segmentectomy, was needed due to the positive intraoperative frozen lymph node examination, while in one case, a lobectomy was performed to ensure negative surgical margins; see Table 2. All surgeries were performed through a triportal VATS anterior approach according to Copenaghen. In all the cases, fissures were completed using automatic mechanical staplers, with a median number of 4 (IQR1-3 3–9) charges used. Skin to skin operative average time was 142 (IQR1-3 105–182.5) min. We had no intraoperative complications.

Among the postoperative parameters, we registered a median of 680 (IQR1-3 415–925) mL of total liquid leaks from the chest drain; the median duration of required thoracic drainage was of 4.4 (IQR1-3 3–6) days, with 1.9 (IQR1-3 0–3.5) median days of air leak; one patient had prolonged air leaks (PAL, defined as lasting longer than 7 days) as a postoperative complication, treated conservatively and without the need of re-intervention. Discharge from hospital occurred on the 6th (IQR1-3 3.5–7) postoperative day (Table 2). Final pathological reports noted lung adenocarcinoma in eight cases, metastases from other neoplasms in two cases, and one lung neuroendocrine tumor (atypical carcinoid).

The median value of the closest surgical margin was 5 (IQR1-3 4–19) mm. According to the VIII edition of the pathological Tumor Node Metastasis (pTNM) classification, primitive lung tumors were in stage I in six cases, stage II in two cases, and stage III in one case.

## 4. Discussion

The emergence of video-assisted thoracoscopic surgery and its integration in the era of new technologies has completely revolutionized the world of thoracic surgery. Moreover, recently, thanks to the minimally invasive trend, the role of VATS segmentectomy has been growing in the treatment of some early stage NSCLC due to its proven efficacy in terms of oncological outcomes and satisfactory post-operative results [4,5,6]. It represents a more complex procedure compared to lobectomy and requires a deep knowledge of the anatomy of the lung, especially because of different possible patterns of anatomical variations. Furthermore, not all the segments are equal; in fact, from a technical point of view, segmentectomies can be categorized as either simple or complex based on the number of intersegmental planes to cut (more than one indicates a complex type). For this reason, complex segmentectomies are notoriously more challenging and technically demanding procedures compared to simple segmentectomies [14]. Moreover, some vascular variations are usually not identified in chest CT scan images, and surgeons may face them only during the surgical procedure, sometimes leading to technical errors. The development of new technologies and their integration in the medical field has given us new tools in understanding each patient’s lung anatomy, thereby assuring the accuracy and the safety of the operation. Furthermore, 3D reconstruction images can serve as an important aid, both before and during the surgical procedure, allowing suitable pre-operative planning.

In this preliminary report, a careful pre-operative evaluation of 3D CT images was performed. In this way, our surgical team could clearly see the precise localization of the tumors, detect the correct segments to remove, and identify pulmonary bronchial and vessel branches with all the Pulmonary Artery (PA) and Pulmonary Vein (PV) branching patterns related to the surgery with precision. In other words, we were able to ‘simulate’ the operation by individualizing the sequence of bronchial and vascular structures to cut.

Each patient was preliminarily evaluated by an expert surgeon, i.e., one having performed more than 100 pulmonary anatomical VATS procedures as the first operator. The evaluation consisted of giving the indication for a segmental resection through a careful visualization of chest CT scan images. The indication at this step was made in the presence of a lung nodule, suspicious or with a diagnosis of malignancy, with a diameter of less than 20 mm and with the absence of enlarged lymph nodes. After that, the necessity of a 3D reconstruction as an aid for the resection was considered. During this process, we proposed the use of a questionnaire to the expert surgeon in which they presented their indication, either after the visualization of 2D images (conventional chest CT scan) or after evaluation of the 3D lung model. The final decision of the type of resection was thus based on the 3D model. In some cases, the indication of the type of resection was changed after examining the reconstructions, indicating their relevance. In other cases, the indication was confirmed using both methodologies. This approach to lung resections is new according to the recent literature, but we found it useful for the completeness of the preoperative evaluations of the patients. Moreover, each case was evaluated by a multidisciplinary team with radiologists and anesthesiologists; in this way, the appropriateness and feasibility of the planned operation could be discussed from a functional and physical point of view in order to guarantee a tailored surgery for each patient.

In our opinion, these notions may have some important clinical implications; firstly, they can helps surgeons to give a proper indication of the type of segment to remove with respect oncological standards and surgical margins. In fact, nowadays, a radical resection margin is considered to be a distance of at least 2 cm from the nodule or a distance of at least that of the major axis of the nodule if it is less than 2 cm [15]. Secondly, a visualization of the anatomical relationship of the reconstructions can serve as a sort of mental training for each operation, thereby preparing surgeons for real surgery. This may reduce the rates of intraoperative complications, making surgical procedures safer. Indeed, different studies have demonstrated an accuracy of up to 98% for surgically pulmonary artery branches, whereby uncommon variations in bronchovascular branching patterns are shown [16,17,18].

In all the procedures, we did not experience any intraoperative complications. The mean operative time was of 142 (IQR1-3 105–182.5) minutes, and no conversions to thoracotomy were required. The median postoperative chest tube duration and discharge day were of 4.5 (IQR1-3 3–6) and 6 (IQR1-3 3.5–7) days, respectively.

The median nodule size was 20 mm (IQR1-3 17–23), with a median surgical disease-free margin of 13.7 mm. Nodules > 20 mm in diameter were evaluated for segmentectomy only in case of metastasis from other cancers, where a wedge resection was not feasible because it could not guarantee a radical resection considering the depth of the nodule. With a median follow up of 12 months, we had no locoregional recurrences, and only one patient died due to a metastatic melanoma two months after intervention.

In this set of 11 patients, 3D CT reconstructions were used to devise a preoperative plan of nine complex and two simple segmentectomies. As said before, the main difference between these two types of segmentectomy was based on the number of segmental planes to cut. The surgical resections planned during the preoperative evaluations were effectively performed in seven of the eleven patients (63%). This may, however, be a misleading result; in fact, only in one case was this related to a discrepancy between 3D CT reconstructions and intraoperative findings. Indeed, in one planned S1–S2 left segmentectomy, at the moment of cutting the intersegmental plan with the lingula, we found that the tumor was too close to the surgical margin, so we decided to perform a left upper lobectomy in order to ensure oncological radicality. Meanwhile, in the other three cases (two complex and one simple segmentectomy), we decided to perform the respective lobectomy based on our intraoperative findings regarding pathological lymph nodes. In fact, as the guidelines suggest [19], in all the segmentectomies, we recommend starting with radical hilomediastinal lymphadenectomy, sending to frozen section evaluation all suspicious N1 and possible N2 stations. In one apical segmentectomy of the right upper lobe (S1 resection) and in one apical segmentectomy of the left lower lobe (S6), a hilar (station #10) and an infrascissural (station #11) metastatic node, respectively, were found in the frozen section examinations. On this basis, we changed our indication and finally decided for a right upper and left lower lobectomy, respectively. Meanwhile, during a right S8–S9 segmentectomy, an intraoperative finding of subcarinal PET-negative adenopathy (station #7) was the basis of the decision to perform a right lower lobectomy, considering the N2 single station metastasis. These findings were totally unpredictable. Indeed, in all three cases described, a careful preoperative stadiation was performed with the three patients; these were then studied with a thin-section contrast enhanced Chest CT scan and a whole body PET-CT scan. Both exams did not show any enlarged lymph nodes or any avidity to fluorodeoxyglucose (FDG), so considering the tumor size less than 2 cm, patients were first enrolled for segmentectomies.

As we know, although 3D-CT reconstructions have demonstrated several advantages, they cannot predict unexpected nodal metastases, so in these three cases, the changing of the surgical indication was not related to preoperative plan errors. Meanwhile, the first case presented suggests that despite the great accuracy and utility of 3D CT reconstructions, surgical skills and experience remain crucial for the final decision regarding the type of lung resection to perform. This is true especially when facing intersegmental planes that in 3D-CT reconstructions are based on a “virtual” reproduction of the supposed pertinence zone of the bronchial branches. To overcome intersegmental related problems, in our opinion, the integration of 3D images with green indocyanine plane identification may provide the most detailed description of the proper margins to be dissected and would likely ensure the best results, especially in complex segmentectomies. In fact, different techniques have been described for the identification of the intersegmental plane. Some examples are the inflation/deflation test after the occlusion of the target segmental bronchus or the inflation of the target segmental bronchus only. However, in several studies, the systemic injection of IndoCianine Green (ICG) has provided an accuracy of over 80%. In particular, Yotsukura and colleagues showed a precision rate of 88% for an ICG group compared with 78.7% when using other techniques like High Frequency Jet Ventilation (HFJV) [20].

In our opinion, the systematic use of 3D-CT reconstructions, both in pre-operative and intraoperative settings, can reduce not only the likelihood of intraoperative complications related to interpretations of segmental anatomy but can also help to decrease operative time, because surgeons have already clearly “seen” the bronchovascular anatomy before surgery. We reported no intraoperative complications, and the surgical time of our preliminary tests were in line with literature results for segmentectomies performed with 3D reconstructions [21]. Nevertheless, we must remember that this paper reports only our preliminary findings, and improvements are ongoing.

Another important advantage of 3D CT reconstruction is the educational role it can play for the training of young surgeons, especially in residency programme. In our opinion, this is probably an underestimated benefit; indeed, with the advent of minimally invasive thoracoscopic surgery and the more widespread use of lung sparing resections, perfect knowledge of lung anatomy is the secret to perform correct and accurate surgical interventions. However, as previously described, lung variations are more common in segmental and sub-segmental anatomies, and they would be difficult to recognize, even for expert surgeons. With 3D images, aspiring surgeons may familiarize themselves with lung anatomy and could approach segmental structures and variations in an interactive way, simulating surgical procedures by cutting the bronchovascular structures in the same order as they would in real surgery. Moreover, there is already evidence that 3D images might improve surgical residency training [21].

Finally, 3D CT reconstruction has some disadvantages, notably, its high cost. Additionally, it requires a significant amount of time and a specific contrast-enhanced CT scan protocol to elaborate images. Nevertheless, recent evidence has noted advancements in reconstructive software, thereby reducing processing time.

Our study had some limitations; firstly, as a preliminary experiment, it included only a small number of patients. Secondly, we analyzed intraoperative and perioperative characteristics only of the 3D reconstruction group. In the future, it would be interesting to compare intraoperative outcomes of segmentectomies performed with or without the support of 3D-CT images. Finally, we would like to emphasize that the principal intent of our study is to demonstrate the safety and effectiveness of the use of 3D reconstructions in segmentectomies from an intra- and postoperative point of view, including also safe surgical resection margins. For this reason, we deliberately included not only primary lung cancers but also lung metastases.

## 5. Conclusions

Despite its technical difficulties, segmentectomy may nowadays be considered a valid alternative to lobectomy for the treatment of NSCLC < 2 cm. Considering this restricted indication, careful and correct pre-operative planning is essential; otherwise, some “surprises” may be still found in the intraoperative setting. With this preliminary report, we confirm the utility and accuracy of 3D CT-guided reconstructions for the correct planning of segmentectomies, reporting a satisfactory concordance rate between the planned and final surgery. However, although, in some particular cases, new technologies may be helpful for surgeons, in our opinion, human skill and surgeon personal experiences still remain fundamental for the final decisions regarding the proper resection to perform.

## Figures and Tables

**Table 1:** Baseline data of the patients and comparison between pre-operative indication and performed surgery.

Patient ID	Sex	Age	CCI	P/y	Tumor Location	Tumor Dimension (CT Scan) [mm]	Pre-Operative Indication	Surgical Intervention	Reason for Changing Indication
1	F	58	7	-	LUL	33	S1 + S2	S1 + S2	-
2	F	45	6	4	RUL	20	S2	S2	-
3	F	63	8	-	LLL	12	S7 + S8	S7 + S8	-
4	F	82	7	-	LUL	34	S1 + S2	LUL *	Ensure negative margins
5	F	62	5	-	RLL	18	Basal pyramid	Basal pyramid	-
6	M	52	4	3	LLL	16	S9 + S10	S9 + S10	-
7	M	66	5	-	RUL	20	S1	RUL *	N1+ frozen-examination
8	M	61	3	18	RUL	21	S1	S1	-
9	F	75	5	-	LLL	25	S8 + S9	LLL *	N2+ frozen-examination
10	M	66	6	50	LUL	11	S1 + S2	S1 + S2	-
11	M	71	5	10	LLL	21	S6	LLL *	N1+ frozen-examination
Median (IQR_1–3_)		63 (59.5–68.5)	5.0 (5–6.5)	0 (0–7)		20.0 (17–23)			

* Cases in which indication changed intraoperatively. CCI: Charlson Comorbidity Index; P/Y: Pack year; LUL: left upper lobectomy; RUL: right upper lobectomy; LLL: left lower lobectomy; RLL: right lower lobe.

**Table 2:** Intra- and post-operative findings for the 11 patients.

Patient ID	Time of Surgery [min]	Number of Parenchymal Charges for the Segmental Plane [n]	Total Liquid Leaks [mL]	Duration of Air Leaks [Days]	Duration of Chest Drainage [Days]	Dismissal [Post-Operative Day]	Pathological Diagnosis	pTNM VIII ed.	StagingVIII ed.	Resectionmargin [mm]	Follow-Up [Months]
1	85	1	760	3	6	7	Adenocarcinoma	T1cN0	IA3	5	9
2	70	3	950	5	6	7	Metastasis	-	-	1	9
3	100	6	100	1	1	3	Adenocarcinoma	T1aN0	IA1	5	12
4	210	5	1480	7	9	10	Adenocarcinoma	T1bN0	IA2	2	24
5	200	4	650	0	3	4	Neuroendocrine tumor	T1bN0	IA2	40	24
6	165	7	500	0	3	5	Metastasis	-	-		5
7	125	3	310	4	8	9	Adenocarcinoma	T1bN1a	IIB	19	11
8	110	4	330	0	2	3	Adenocarcinoma	T2N0	IB		12
9	205	3	900	1	4	5	Adenocarcinoma	T2N2b	IIIA	12	32
10	150	4	850	0	3	4	Adenocarcinoma	T1aN0	IA1	35	34
11	145	4	1300	0	3	5	Adenocarcinoma	T1cN1a	IIB	4	1
Median (IQR_1–3_)	115 (105–182.5)	4 (3–9)	1030 (415–925)	1.5 (0–3.5)	4.5 (3–6)	6 (3.5–7)	-	-	-	5 (4–19)	12 (9–24)

## Data Availability

All data are available upon request from the Corresponding Author.
